# Phenol Neurolysis in Pain and Palliative Medicine

**DOI:** 10.1111/papr.70153

**Published:** 2026-04-13

**Authors:** Jeroen H. A. Creemers, Rachel J. H. Smits, Alopi M. Patel, Robert T. M. van Dongen, Cornelis K. Kramers, Jacqueline M. Bos, Selina E. I. van der Wal

**Affiliations:** ^1^ Department of Internal Medicine CWZ Nijmegen the Netherlands; ^2^ Department of Clinical Pharmacy CWZ Nijmegen the Netherlands; ^3^ Department of Anesthesiology, Pain‐ and Palliative Medicine Radboud University Medical Center Nijmegen the Netherlands; ^4^ Department of Anesthesiology and Perioperative Medicine Rutgers‐Robert Wood Johnson Medical School New Brunswick New Jersey USA; ^5^ Department of Pain Medicine CWZ Nijmegen the Netherlands; ^6^ Department of Pharmacology and Toxicology Radboud University Medical Center Nijmegen the Netherlands

## Abstract

**Background:**

Pain is a common symptom in palliative care and affects patients' quality of life considerably. Standard analgesics are sometimes insufficient and are associated with substantial side effects. Neurolysis, the targeted destruction of nerves using thermal or chemical agents, offers an additional option for managing localized pain in palliative care. Phenol, a widely available chemical neurolytic agent known for its painless injection and hyperbaric properties when dissolved in glycerin, can effectively relieve refractory cancer pain.

**Methods:**

A literature review was conducted on the pharmacology, toxicology, and indications of phenol in interventional pain and palliative medicine.

**Results:**

Despite its use as a neurolytic agent for nearly a century, our current understanding of phenol's pharmacological properties, toxicity, and efficacy stems mainly from case series and small studies, often published decades ago. To date, no uniform guidelines exist, nor is there consensus on the optimal application of phenol in clinical practice, resulting in variability among practitioners. Chronic pain syndromes affect up to 30% of the global population, prompting the expansion of phenol neurolysis to new applications, such as joint denervation and sympathetic blocks in non‐cancer pain. Understanding the mechanism of action of phenol and standardizing its clinical use are crucial for integrating this technique into broader pain management strategies.

**Conclusion:**

This review provides a comprehensive overview of the mechanisms, indications, benefits, and safety of phenol neurolysis in pain and palliative medicine, aiming to support its evidence‐based use in clinical practice.

## 1 Introduction

Pain is one of the most common symptoms affecting the quality of life in patients in palliative care [1, 2]. In up to 30% of these patients, standard analgesic treatment is insufficient [3]. Management of these patients is based on a multimodal approach [4]. Neurolysis can be an additional strategy for pain relief in localized pain syndromes, not only in palliative care but in chronic non‐cancer pain as well [3, 5]. Neurolysis is the targeted destruction of sensory and autonomic nerves and can be performed using chemical or thermal agents. It can be applied in sympathetic, peripheral nociceptive, or intrathecal blocks. The most commonly used thermal techniques include radiofrequency ablation or cryoablation [6].

Chemical neurolysis is primarily used as an adjuvant for the treatment of refractory cancer pain, reducing the consumption and, therefore, the side effects of other analgesics [7]. Given its anecdotal effect duration of 1–6 months and the suggested risk of developing pain and neurological deficits due to neuritis, chemical neurolysis is predominantly used in end‐of‐life care [8, 9]. The risks associated with a chemical neurolytic block primarily depend on the interventional technique and anatomical location. Logically, when motor nerves are involved, the estimated beneficial effect on pain relief must be carefully weighed against the possibility of additional motor impairment.

**FIGURE 1:**
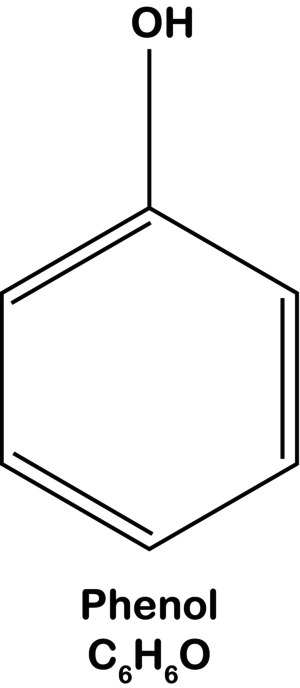
Structural formula of phenol.

The neurolytic agents used in daily practice are ethanol and phenol [10]. Phenol, also known as carbolic acid, is composed of a benzene ring in which a hydroxyl group substitutes one hydrogen atom (Figure 1). Phenol can be dissolved in either water or glycerin, and the choice of solvent affects its application. When dissolved in glycerin, phenol becomes hyperbaric compared to cerebrospinal fluid (1.26 g/cm^3^ and 1.006 g/cm^3^, respectively; see Box 1). This property is particularly relevant in intrathecal applications, where the hyperbaric solution can be gravitationally directed using positioning to target specific dermatomes. In contrast, phenol in water is not hyperbaric and has a lower viscosity. Therefore, it is typically used for peripheral or sympathetic nerve blocks. Compared to ethanol, phenol is painless upon injection. The neurolytic effects of phenol are achieved in approximately one to a few days. However, a drawback is that phenol is less widely available than ethanol; as an advantage, ethanol does not require a solvent [11].

BOX 1 Phenol neurolysis can be performed using phenol dissolved in either water or glycerin. The concentration of phenol is typically expressed as a mass/volume percentage $\frac{\text{phenol}\;\left(\mathrm{mg}\right)}{\text{volume}\;\left(\mathrm{mL}\right)}\times 100\%$. For example, 6% phenol solution in water solution corresponds to 60 mg of phenol per milliliter of water. When available, the specific solvent is reported.

Chronic pain has a high disease burden and affects more than 30% of the global population. Although not considered standard‐of‐care for non‐cancer pain, phenol neurolysis is increasingly used to treat visceral pain syndromes through sympathetic blocks and localized pain by predominantly targeting sensory nerves [12]. Examples include procedures such as splanchnic blocks in chronic visceral pain syndromes or ganglion impar blocks in coccygodynia [13]. Moreover, it is an increasingly used technique in joint denervation, offering the potential benefit of more extensive neurolytic fluid spread in sites with high anatomical variation [14, 15, 16, 17].

Phenol has been used for nearly a century and is still part of daily pain practice, yet a clinical guideline addressing its use and safety concerns remains unavailable. This review describes the mechanism, indications, benefits, and safety of phenol neurolysis in pain and palliative medicine.

## 2 A Brief History of Phenol and Its Use in Neurolysis

Phenol was first discovered in its impure form in 1834 by Runge [18]. He isolated phenol from coal tar, a byproduct of coal gasification, and named it “Karbolsäure” – carbolic acid. In 1841, Laurant successfully extracted phenol in its pure form [19]. Its first medical application followed in 1867 when Joseph Lister introduced it as an antiseptic in surgical procedures. Lister noted phenol's “peculiarly destructive influence upon low forms of life” [20]. This observation played a pivotal role in the development of aseptic surgical procedures. Over half a century before phenol's use in neurolysis, Luton described the first use of non‐phenol neurolytic agents in 1863 [21]. Phenol as a neurolytic agent was first used by Doppler in 1926 in animal studies [22]. In 1945, Boll employed phenol for matrixectomy in the treatment of ingrown toenails [23]. In 1950, Mandl reported its use in sympathetic ganglion block in animals, implying permanent sympathectomy [24]. Maher first used phenol in a hyperbaric solution for intrathecal injection in humans in 1955, marking a pivotal step in managing pain and spasticity [25].

## 3 Phenol's Neurolytic Action: A Pharmacological and Toxicological Perspective

### 3.1 Pharmacokinetics

Research on the pharmacology and toxicology of phenol has primarily involved animal studies and case reports, focusing on oral and inhalational exposure. However, these findings can be extrapolated to peripheral, visceral, or neuraxial blocks. For a detailed overview of the pharmacokinetics of phenol, we refer to reference [26].

Phenol is rapidly absorbed into the systemic circulation. After injecting 5–10 mL of 7% phenol for lumbar or thoracic sympathetic blockade, unconjugated phenol enters the systemic circulation within 5 min, reaching a peak concentration (*C*_max_) of 3.01 mg/L after approximately 19 min [26].

Although studies on phenol distribution are available, they primarily involve dermal, inhalation, or oral administration routes. At the same time, distribution data after sympathetic, peripheral, or intrathecal injection in mammals is scarce. Given its lipophilicity, phenol is expected to have a large volume of distribution regardless of the route. Animal studies, mostly in rats, have shown that when phenol is administered through the skin, inhaled, or ingested, it distributes widely across the body, reaching the blood, skin, muscle, bone, fat, and visceral organs [27, 28].

The primary metabolic pathway for phenol, elucidated primarily through studies in animal models, involves direct conjugation with sulfate or glucuronic acid either via phenol sulfotransferase or UDP‐dependent glucuronosyl transferase in the liver. At low doses, sulfation predominates [29]; this dose‐dependent pattern is thought to arise from the depletion of the sulfate pool and differences in K_m_ values for sulfate and glucuronide conjugation [30, 31]. Preclinical data indicate a switch to glucuronidation from systemic doses of 12.5 mg/kg onwards. At higher doses, oxidative metabolism—primarily via CYP2E1—increases, as reflected by urinary analyses in tracer studies using ^14^C‐phenol in mice. After intraperitoneal administration of 75 mg/kg phenol (*C*_max_ 38.6 ± 8.2 mg/L in blood after 5–10 min), urinary metabolites consisted of phenyl sulfate, phenyl glucuronide, and hydroquinone glucuronide, with each contributing for approximately 30% of the administered dose [32, 33]. As CYP2E1 blocking antibodies inhibit only two‐thirds of hydroquinone formation, the involvement of other P450 enzymes—such as CYP2F2—is suggested [34, 35]. Moreover, in vitro experiments suggest that CYP2E1 can convert catechol and hydroquinone into trihydroxybenzene prior to their sulfation and glucuronidation. Additionally, hydroquinone may undergo peroxidation to form benzoquinone, followed by glutathione conjugation [36]. Isolated perfused liver experiments in rats show a dose‐dependent first‐pass metabolism. At low phenol concentrations (e.g., 0.05 mg/L), hepatic extraction reaches approximately 98%. However, as concentrations increase (e.g., 2.9, 26, and 240 mg/L), hepatic extraction decreases to 73%, 26%, and 5%. In serum, phenol is 52.7% protein‐bound [28].

Phenol and its metabolites are primarily excreted in urine. A minor portion (estimated at < 5%) is excreted via bile. Estimates suggest that after a sympathetic block injection with phenol 5–10 mL 7% in water, the half‐life of phenol is approximately 30 min. Conjugated phenol reaches a *C*_max_ of 4.15 mg/L after 55 min and has a half‐life of 65 min [26]. It remains unclear to what extent the solvent affects the pharmacokinetics of phenol.

### 3.2 Pharmacodynamics

Phenol exhibits a dose‐dependent and non‐selective cytotoxic effect [11, 37]. Initially, phenol was thought to affect only smaller nerve fibers (Aδ, Aγ, and C fibers); however, studies in 1963 revealed that it non‐selectively damages nerve fibers, regardless of their diameter or myelination status. At low concentrations (i.e., 1%–2%), phenol acts as a local (topical) anesthetic agent [37]. The optimal concentration of phenol as a neurolytic is not well‐studied and ranges from 3% to 12%, with most evidence suggesting demyelination occurs at concentrations above 5%. Although the exact biochemical mechanism leading to chemical denervation remains to be elucidated, phenol is presumed to induce protein coagulation and denaturation. Phenol injection near neural tissue causes demyelination and axonal edema [6]. Whether nerve cells are more susceptible to phenol than other surrounding cells remains to be elucidated.

The clinical effect of phenol is limited by neuronal regeneration, with analgesia lasting approximately 2 to 12 months [38]. Primary sensory neurons can extensively regenerate axons, more so in peripheral than in central axons. Besides the neuron's intrinsic regeneration capability, this may be attributed to a favorable environment of the surrounding tissue for axonal growth. Axonal regeneration begins within 24 h post‐lesion, followed by macrophage infiltration within 2 to 3 days [39]. Regenerating axons cannot select their original endoneural tubes, potentially leading to altered reinnervation [40]. Dysesthesia due to this effect or neuritis is a feared risk, with the dysesthesia or hyperalgesia potentially being worse than the initial pain [6]. However, a large case series on ultrasound‐guided peripheral nerve blocks with phenol in spasticity reported zero to < 1% cases of dysesthesia [41, 42, 43].

In rats, intrathecal phenol application resulted in more damage to the central roots than to the peripheral roots, primarily due to a thrombotic effect in the vessels peaking at the injection site. Macrophages were absent after 2 days but were present by Day 14. Axonal sprouting was observed after 2 weeks, followed by remyelination at 2 months [44]. Dysesthesia is more common after intrathecal administration, with a reported complication rate of 8% in case series [45].

### 3.3 Toxicology

For an extensive overview of the toxicological data on phenol, we refer to references [27, 36, 46]. Toxicological data on phenol is mainly derived from animal studies or case reports involving oral, dermal, or inhalational exposure. Limited data are available regarding the toxicity following phenol neurolysis. Toxic serum phenol levels following dermal or oral exposure range from 1 to 130 mg/L; whether this refers to free phenol or total phenol (free plus bound) is not specified [47]. Interpreting these data presents challenges, including uncertainties in serum sampling timing, exposure volume, and assessment of phenol's free or protein‐bound fraction. Human lethal oral dose estimates for phenol vary, ranging from approximately 14 to 930 mg/kg in adults [27]. Given its rapid distribution, phenol toxicity is theoretically expected to occur in a time window ranging from minutes to an hour [48]. Here, we limit our focus to acute phenol toxicity.

Phenol has hydrophilic and hydrophobic properties. This amphiphilic character enables it to cross cell membranes easily, a property crucial to its toxicological profile. Given that its mechanism of action involves protein degradation, local toxicity may manifest as coagulation necrosis. Moreover, dermal exposure can induce a dose‐dependent range of symptoms, from painless hypopigmentation—due to selective damage to melanocytes and competitive inhibition of tyrosinase during melanin formation—to erythema, skin blistering, and local necrosis. Repeated dermal exposure has been reported to induce vitiligo as well as hyperpigmentation. The pathophysiology behind this ambiguous dermal response is not yet fully understood. Local toxicity following ingestion of phenol includes corrosive lesions to the upper gastrointestinal tract.

Systemic toxicity in humans can be induced via any route of administration and manifests itself through a plethora of symptoms for which the exact mechanism of action is often not completely understood. The central nervous system (CNS) involvement, marked by an initial transient excitation phase followed by depression and eventually coma, is the primary cause of death from phenol toxicity. Other CNS‐related symptoms encompass headache, dizziness, seizures, and extrapyramidal effects (including the “rabbit syndrome,” an antipsychotic‐induced rhythmic motion of the mouth and lips) [49, 50]. Hematologic toxicity includes hemolysis and formation of methemoglobin [51]. Cardiovascular toxicity manifests as initial hypertension, followed by hypotension and shock. Ventricular arrhythmias leading to cardiac arrest, potentially mediated via phenol's blockade of cardiac sodium channels, have been described [52, 53, 54, 55, 56]. Hypotension contributes to renal failure via acute tubular necrosis and is frequently observed [57]. Additionally, direct toxic effects of unconjugated excreted phenol are suggested to induce glomerular damage, possibly through free radical intermediates or epithelial cells' inability to form reduced glutathione to clear phenols' intermediates. Furthermore, phenol‐induced hemolysis contributes to hemoglobin cast nephropathy [58, 59]. While postmortem pharmacokinetic data is scarce, the renal phenol concentration in a fatal poisoning case was found to be 4–8× as higher than other organs, potentially contributing to its nephrotoxic effects [60]. Other fatal poisoning cases show different distribution profiles without renal predilection [61]. Rhabdomyolysis and hemolysis‐induced toxic Hb dimers might further deteriorate renal function [48, 51, 62]. Gastrointestinal symptoms of systemic toxicity include nausea, vomiting, diarrhea, and abdominal pain [49]. Metabolic acidosis following phenol poisoning, believed to result from phenol interfering with cellular respiration—specifically, inhibition of Complex III of the mitochondrial electron transport chain—is described [47, 57, 63]. It remains unclear whether the acidosis is attributable to tubule dysfunction, an anion‐gap metabolic acidosis driven by lactate accumulation, or a combination of both factors. Respiratory toxicity includes hypoxia resulting from acute respiratory distress syndrome [59].

Regarding pregnancy and lactation, data on (the safety of) phenol and phenol neurolysis is not available. Animal studies suggest that environmental exposure at levels toxic to the mother might induce congenital disorders, but human data is unavailable [49]. For further toxicity‐specific information on patient history, physical examination, differential diagnoses, and toxicity management, we refer to [48, 49].

## 4 Indication(s) for Phenol Neurolysis in Pain Management

### 4.1 Neuraxial Neurolysis

Neuraxial neurolysis—the destruction of nerve pathways within the spinal axis—with phenol places demands on the solvent. Phenol in glycerol is a hyperbaric liquid relative to cerebrospinal fluid and facilitates the gravity‐dependent targeting of nerve roots by positioning the patient. It has a high viscosity and requires larger bore needles for injection [45]. Warming phenol in glycerol to body temperature might ease the injection due to the significant rise of the viscosity at lower temperatures [64].

Titration of the volume is advised, starting at 0.2 mL or with 0.4 mL per dermatome [10, 25, 65]. The concentration varies from 3% to 12%, with 5% to 10% as most often used with a recommended concentration of 5%–6% to limit side effects (e.g., axonal abnormalities, spinal cord infarct, arachnoiditis, meningitis) [45]. Fluoroscopy can be used to confirm the targeted site. When successful, neurolytic intrathecal blockage provides immediate pain relief. However, it also causes motor weakness and numbness and can lead to bladder and bowel dysfunction. It is described in recent literature in patients with inoperable femur fractures unfit for surgery as pain relief [66, 67, 68]. No randomized controlled trials have been published; the literature primarily comprises case reports and retrospective cohorts [45, 69]. Neurolytic saddle blocks or lower‐end blocks can be performed for intractable pelvic, sacral, or perineal pain [70]. Epidural injection of phenol has been described as pain relief due to lytic lesions. The technique is known for its lower complication rate but is used less frequently due to variability in dermatomal spread. Administrated volumes range from 0.5 to 5 mL phenol 3%–12%—often dissolved in glycerin [45, 65].

### 4.2 Sympathetic Neurolysis in Palliative and Cancer Care

The most well‐known application of neurolysis with phenol for managing intractable cancer pain involves sympathetic nerve blocks—including the coeliac plexus, splanchnic nerves, inferior and superior hypogastric plexus, and ganglion impar blocks. Table 1 provides an extensive overview of the literature on phenol neurolysis in cancer pain.

**TABLE 1:** Overview of the literature on phenol neurolysis in cancer pain.

Author (year) [ref]	Procedure	*N*	Formulation (concentration phenol; solvent; volume)	Findings	Safety^a^
Abdelghaffar et al., (2019) [71]	CPB	34	10%; contrast; 25/12.5 mL phenol, 5/2.5 mL contrast	Lower failure rate and shorter treatment duration with single needle (*n* = 17) than double needle (*n* = 17) approach; marked pain reduction	Hypotension, diarrhea, vomiting, hemorrhage, infection (equal distribution)
Abdelghaffar et al., (2022) [72]	SHPB for pelvic pain	96	10%; ND; 20 mL + 3 mL 5% bupivacaine	Comparable NRS decline in US‐guided (*n* = 48) and fluoroscopy‐guided (*n* = 48) groups	No serious complications
Ahmed et al., (2015) [73]	SHPB + GIB for pelvic/perineal pain	15	10%; saline; 10 mL + 8%; saline; 4–6 mL	Marked pain reduction; less morphine consumption	No complications or serious adverse events during/after the block
Antila & Kirvelä, (1998) [74]	Paravertebral block	7	7%; water; 1–4 mL	Varied pain relief duration (poor to > 4 months)	No complications
Erdine et al., (2003) [75]	SHPB transdiscal approach for pelvic pain	20	10%; water; 5 mL	Marked pain reduction	No complications
Ischia et al., (1984) [76]	SAB (L5‐S1) for pelvic pain	33	7.5/10/15%; glycerine; 0.6–1 mL	50%–60% complete, 20%–30% partial pain relief	No complications
Ishiwatari et al., (2014) [77]	CPB for upper abdominal pain	22	7%; water; 20 mL (max.) or Absolute ethanol	Marked pain reduction; response rate at Day 7: phenol 83% (*n* = 6), ethanol 69% (*n* = 16)	Diarrhea (*n* = 1; in phenol group)
Koyyalagunta et al., (2016) [78]	SNB for upper abdominal pain	93	10%; glycerine 20%; ND or 98% dehydrated ethanol	Comparable pain reduction between phenol (*n* = 67) and ethanol (*n* = 27); ~45% had ≥ 30% pain reduction and ~44% no pain reduction	Hypotension (*n* = 2); weakness in leg (*n* = 1; unrelated); shortness of breath (*n* = 1)
Malik et al., (2018) [79]	Transcrural CPB	35	6%; ND	Marked pain reduction; reduced analgesic use	
Matchett, (2016) [80]	Intercostal nerve block for chest wall pain	11	8.9%; mixture; 2–3 mL	Marked pain reduction (4/6 patients)	No complications
Mohamed et al., (2013) [81]	Transsacral IHPB	20	10%; saline; 6–8 mL (bilateral)	Marked pain reduction; less morphine consumption	No complications; transient paresthesia (*n* = 7), pain on injection (*n* = 4), vascular penetration (*n* = 1)
Nagaro et al., (1994) [82]	SAB	13	10%; glycerine; 0.2–0.3 mL	Marked pain reduction; less morphine consumption	No complications
Rahman et al., (2018) [83]	Retrocrural CPB for upper abdominal visceral pain	507	6%; water; 3–5 mL	Marked pain reduction; improved QoL; reduced opioid use	Diarrhea (*n* = 77); orthostatic hypotension (*n* = 1)
Robertson et al., (1983) [84]	GIB for perineal pain	9	6.66%; water; 2.5 mL	Satisfactory immediate effects; 7 patients required further treatment ≤ 10 days	No complications
Rocha et al., (2020) [85]	SHPB for pelvic pain	180	10%, water, 8–10 mL (bilateral)	Pain reduction VAS 5.7 to 0.9 at 24 h, pain relief persisted 6 months in 88/180	Transient hypotension (*n* = 10), transient urinary incontinence (*n* = 1), hypertension (*n* = 1)
Rodriguez‐Bigas et al., (1991) [86]	Intrathecal block (L3‐4/L4‐5) in rectum carcinoma	11	450 mg phenol in 5 mL iofendylate	Good (2–10 months; *n* = 3) to fair (*n* = 3) pain relief	No complications
Salmon et al., (1992) [87]	Epidural block	15	5%; glycerine; 2–4 mL	Marked pain reduction	No complications
Slatkin et al., (2003) [70]	Intrathecal saddle block for pelvic pain	4	6%; glycerine; 0.6–1 mL	Marked pain reduction	Temporary leg weakness in 2/4 patients
Tei et al., (2008) [88]	Mixed population	≥ 30	ND	Phenol‐specific findings are not reported. Groups: intrathecal block (*n* = 21), epidural block (*n* = 9)	Adverse events for phenol are not specified
Toshniwal et al., (2007) [89]	GIB	16	8%; water; 4–6 mL	Marked pain relief during 2 months follow up (VAS after 2 months: 2)	No adverse events
Turker et al., (2005) [90]	SHPB via transdicsal approach	3	10%; ND; 8 mL	Marked pain reduction up to 6–12 months	No early or late complications
Varghese et al., (2001) [91]	Endoscopic transnasal neurolytic SPGB	22	6%; water; 0.5 mL	Complete (*n* = 17) and partial (*n* = 1) pain relief	Postprocedural nausea and giddiness (*n* = 1)
de Leon‐Casasola et al., (1993) [92]	SHPB for pelvic pain	26	10%; water; 8 mL (bilateral)	Satisfactory pain relief (*n* = 18); moderate pain control (*n* = 8); reduced opioid use	Transvascular neurolytic block (*n* = 2); Burning sensation (*n* = 1)

Abbreviations: CPB, Celiac plexus block; GIB, ganglion Impar block; IHPB, Inferior hypogastric plexus block; SAB, Subarachnoid block; SHPB, Superior hypogastric plexus block; SNB, Splanchnic nerve block; SPGB, sphenopalatine ganglion block.

^a^ Phenol‐related.

By blocking visceral nociception, a neurolytic block of the coeliac plexus can provide pain relief for patients with upper abdominal malignancies (e.g., pancreatic cancer). It leads to better pain control, reduced opioid consumption, and less constipation. Moreover, the procedure can be performed either by surgical resection or through a fluoroscopic or CT‐guided approach, administering 10 mL of neurolytic fluid on each side [65, 83]. Another option for pancreatic cancer‐related pain is a bilateral splanchnic nerve block. These blocks are often performed at the Th11 level combined with blocks at Th10 or T12. The advised dose per approach or level is an injection of 4–8 mL of phenol 6%–10% [93]. In cancer‐related pelvic pain, several techniques can be used to target the superior hypogastric plexus. When contrast spread is bilateral with one needle, 15 mL of 6%–10% aqueous phenol is applied, or 8–10 mL per side if two needles are used [85, 93]. The inferior hypogastric plexus is a target for neurolysis in both pelvic and perineal cancer‐related pain. Via a trans‐sacral approach, 6–8 mL of phenol 10% can be injected bilaterally for pain reduction [81]. The most distal sympathetic neurolytic block is the ganglion impar block. The sympathetic chain at the level of the coccyx can be targeted with phenol to alleviate visceral pain caused by perineal tumors. The needle is placed through the sacrococcygeal ligament, and 4–6 mL phenol 10% is injected to target the ganglion [65]. Combining neurolysis of the ganglion Impar and superior hypogastric plexus is also described, using a total of 14–16 mL of 10% phenol in saline—10 mL for the hypogastric and 4–6 mL for the ganglion Impar [73]. For refractory pain in advanced head and neck cancer, neurolysis of the sphenopalatine ganglion with 1 mL phenol 6% is described [94].

### 4.3 Sympathetic Neurolysis in Chronic Pain and Other Implications

Besides cancer and palliative care, sympathetic blocks with phenol are used for other indications [10]. They are mainly used in interventional treatment strategies for patients with complex regional pain syndrome (CRPS) [95]. Neurolysis of the sphenopalatine ganglion is applied in patients with head and facial pain. Apart from a case series using 88% phenol intranasally to treat atypical facial pain, no other phenol infiltrations are described for these conditions. In these cases, a tightly wrapped cotton carrier dipped in 3 cc of 88% phenol (solvent not mentioned) is applied to the sphenopalatine ganglion for 15–30 s up to 5 times [94, 96, 97]. For patients with CRPS, vascular compromise, or neuropathic pain in the upper extremities, neurolysis of the sympathetic chain at the Th2 and Th3 levels, involving the injection of 2 mL of 6%–10% phenol at each level, has been described. It is also described in sympathetically maintained persistent pain after stellate ganglion neurolysis [93, 98]. Splanchnic neurolytic nerve blocks can also be considered for chronic pain syndromes, such as chronic pancreatitis. They can be performed at the Th10, Th11, and Th12 levels, as described above [6, 99, 100]. Neurolysis of the lumbar sympathetic ganglion is described in CRPS, ischemic pain, and erythromelalgia. Bilateral injection of 2–3 mL of 5%–7% phenol per level led to pain reduction [4, 101, 102, 103]. A pilot study involving 10 patients reported one instance of post‐sympathectomy neuralgia after lumbar sympathetic neurolysis [102].

### 4.4 Peripheral Nerve Blocks

Peripheral neurolytic nerve blocks are used to manage intractable, localized cancer pain. In the treatment of head and neck cancer, neurolysis involving the injection of 1 mL of 6% aqueous phenol per nerve or nerve branch is described for the trigeminal nerve (V2 and V3 branches), occipital nerve, and glossopharyngeal nerve [104]. Neurolysis can also target pain caused by tumors localized in the extremities or invading the peripheral plexuses. Case reports describe brachial plexus neurolysis using 10 to 20 mL of 6% phenol through various approaches, resulting in loss of sensation and motor weakness [105, 106]. Neurolysis of the lumbosacral plexus, using 3 mL of 5% phenol in glycerin, is reported to be successful [107]. The chest wall and thorax are other possible targets. Intrapleural and erector spinae plane blocks with 10 to 12 mL phenol 6%–10% were reported [65, 108]. A commonly used technique involves neurolysis of the intercostal nerves, administering 2 to 3 mL of 6% aqueous phenol per level [80].

Neurolysis of peripheral nerves is also described for treating chronic non‐cancer pain, primarily targeting sensory nerves without affecting motor function. Phenol neurolysis has been described in groin pain (the genitofemoral or ilioinguinal nerve), in meralgia paresthetica (the lateral femoral cutaneous nerve), in occipital neuralgia (occipital nerve), or in painful neuromas [104]. Chemical neurolysis is also getting more common in chronic joint pain due to osteoarthritis. A recent case series described the denervation of the shoulder capsule with phenol for osteoarthritis. For the anterior shoulder capsule, 4 mL of 6% phenol is administered. For the posterior approach, a total of 2 mL is given in increments of 0.5 mL along the posterior glenoid to minimize the spread to motor nerves [109]. For the ablation of genicular nerves in knee osteoarthritis, 6%–7% phenol can be administered at three targets, with 3 mL at each targeted nerve [14, 17]. Another technique involves injecting 0.5–2.5 mL of 6% phenol at five target points [15]. For patients with hip fractures unfit for surgery, chemical neurolysis via denervation of the anterior hip capsule—and the posterior capsule if needed—has been described [110]. The volume of phenol (5 or 6%) ranged from 7 to 10 mL [111, 112, 113].

## 5 Similarities and Differences to Other Neurolytic Treatments

The most commonly used neurolytic fluid besides phenol is ethanol. It is mainly used in 50% or 100% solutions. The neurolytic effect causes non‐selective nervous destruction due to the denaturation of cell membrane proteins, lipid extraction, demyelination, and Wallerian degeneration, meaning the degeneration of the nerve distal from the lesion. Ethanol causes a burning sensation on injection. Ethanol toxicity can consist of dysesthesia or hyperesthesia, tissue necrosis, cardiac rhythm disturbance, vasospasms, hypotension, and central nervous system excitation [114]. Ethanol is less viscous than phenol and acts as a hypobaric liquid in cerebrospinal fluid. It causes pain relief in order of weeks to months and is anecdotally considered to have a slightly longer effect compared with phenol, although randomized controlled studies are lacking. A clinical study on splanchnic nerve neurolysis found no difference in pain relief intensity between ethanol and phenol [78]. With intrathecal use, pain relief was considered good in a similar percentage of patients. A merged population based on case series published between 1950 and 1984 reported fewer complications when ethanol (*n* = 574) was administered intrathecal compared to phenol (*n* = 704) (bladder or rectal sphincter dysfunction 3.5/0.0 vs. 9.0/2.0%, paresis 3.9% vs. 12.9%, dysesthesia 3.8% vs. 8%) [45]. A recent scoping review on neurolysis of the genicular nerves found only mild temporary adverse events after either phenol and ethanol [115].

Other historically used agents are hypertonic saline, glycerol, ammonium salts, chlorocresol, and botulinum toxin A [10]. Glycerol is a naturally occurring alcohol. It is not destructive in concentrations below 50%. It acts as a non‐selective conductive blockade within minutes of application. It also blocks spontaneous activity within damaged axons. Glycerol causes total degeneration of the nerve fibers when injected intraneural. The degeneration might continue for over a week. Compared to phenol, glycerol penetrates the perineurium to a lesser extent with a smaller nerve damage area and theoretically less clinical effect when used perineurally. Regeneration is comparable with phenol, with first axonal sprouts within 1 to 2 weeks, growing larger in 8 weeks with thinner myelin sheaths up to 6 months. Schwann cells increase at 4 weeks, but the number of cells is dependent on intraneural versus perineural injection [116, 117]. The comparison between phenol, ethanol, and glycerol is summarized in Table 2.

**TABLE 2:** Comparison of the most commonly used neurolytic fluids.

Drug (chemical formula; references)	Mechanism of action	Indication	Contra‐indication^a^	Toxicity
Phenol (C_6_H_6_O)	Direct neurotoxicity and ischemia. Denaturation of proteins, loss of cellular fats, separation of the myelin sheath from the axon and axonal edema	Matrixectomy Neurolysis Local anesthetic in HNT Cosmetic peeling	Liver disease (caution)	Extensive overview in text Tinnitus, flushing with intravascular injection Dys‐/hyperesthesia, nausea, and vomiting, CNS stimulation, cardiovascular depression, tissue necrosis
Ethanol (C_2_H_5_OH)	Precipitation of cell membrane proteins, lipid extraction, and demyelination leading to Wallerian degeneration	Neurolysis Disinfectant		Burning pain Tissue necrosis Dys−/hyperesthesia Cardiovascular: vasospasms, rhythm disturbances, hypotension CNS excitation
Glycerol (C_3_H_8_O_3_)	Hygroscopic effect, Wallerian degeneration	Solvent Neurolysis		Tissue necrosis

^a^ On theoretical grounds.

Besides chemical neurolysis, other neuroablative techniques can also be used for targeted nerve denervation, such as cryoablation and radiofrequency ablation. With the advent of these techniques that offer targeted, controlled temperature‐dependent neurolysis, chemical neurolysis has fallen out of favor. However, chemical neurolysis remains the preferred neuroablative modality for certain anatomical regions, such as the abdomen, where temperature‐based ablation techniques are challenging. In joint pathology, phenol has the possible benefit of a more extensive neurolytic fluid spread and targeting more articular branches compared to thermal ablation. Denervation of the shoulder or hip was often achieved using thermal ablative techniques, which selectively create thermal lesions. However, the anatomy of innervation can vary among individuals. The same applies to the denervation of the genicular nerves, where high anatomical variability is reported as well [118, 119, 120]. Peripheral nerves refractory to thermal ablation might be another potential target for phenol neurolysis. Appropriate patient selection based on the specific sensory/motor innervation of the nerve would be necessary to determine the feasibility of phenol neurolysis (i.e., pudendal nerve, obturator nerve) [121, 122]. These considerations render comparative studies between temperature‐based ablation procedures and phenol neurolysis of interest for future research.

Botulinum toxin type A can be an alternative for chemical neurolysis due to its reversible neurolytic effect. It has an antinociceptive effect through the enzymatic blockade of neurotransmitter release, such as acetylcholine, glutamate, and Substance P, providing temporary chemical denervation. It is most known for causing muscle paralysis, which lasts about 3 months [123, 124]. It is mainly used in pain medicine for headache disorders. However, there is rising evidence that it also has an effect for up to 3 months when used in other sympathetic nerve blocks without pathologic cellular changes [125]. Future studies are needed to validate this theory.

## 6 Discussion

Phenol is used mainly as a neurolytic in pain management in cancer pain and palliative care. It is also often used in spasticity, chemical peeling, or matrixectomy. Current knowledge of phenol in pain interventions is mainly derived from case series and retrospective studies. More extensive prospective studies and randomized clinical trials are needed to validate these results. Since clinical guidelines are not widely available, we suggested a practical guideline based on the results of this review (Box S1).

Given the absence of large clinical comparative trials, most knowledge on the effectiveness and possible complications is based on expert opinion and case reports and series. Thorough pharmacokinetic, pharmacodynamic, and toxicologic analysis following neurolytic procedures is lacking, and data on long‐term clinical effects is scarce. For example, phenol is assumed to produce a clinical effect that is less intense and of shorter duration compared to ethanol. Only one clinical trial and a synthesis of case series compare the effect of both agents in pain management, finding no difference in pain intensity [45, 78]. Moreover, several studies on spasticity management demonstrate comparable effectiveness between phenol and ethanol. Additionally, small, non‐randomized trials suggest a greater incidence of phenol‐related systemic side effects, while others show no difference [6].

Although the incidence of side‐effects is not well documented due to the scarce literature, the fear of dysesthesia after the use of phenol does not seem justifiable, with a reported incidence of < 1% [41, 42, 43]. This also holds for ethanol, where the exact incidence of dysesthesia after peripheral nerve neurolysis remains not well established. While a cohort on mandibular nerve neurolysis reported an 11.2% incidence for paresthesia, dysesthesia, or deep sensory loss, other studies do not report any or only transient hypesthesia after peripheral nerve neurolysis with ethanol [126]. To our knowledge, no comparative trials of phenol versus ethanol for peripheral nerve neurolysis have been published.

Intrathecal administration of phenol appears to have a higher risk of dysesthesia, both compared to the use of phenol on peripheral nerves and compared to the intrathecal administration of ethanol. However, the reported incidence of 8% after intrathecal administration is based on several case series between 1950 and 1984 [45]. There is no data on the influence of concentration on these side effects. Soft tissue necrosis—another feared side effect—occurred in one patient with diabetes and peripheral arterial occlusive disease in a series of 156 infiltrated painful neuromas with phenol 80% [127]. Other large case series did not report any signs of necrosis [41, 42, 43].

Phenol can be used as a neurolytic agent in any interventional technique, as there is no established difference in the degree of pain relief between the administration of phenol or ethanol [114]. Being painless on injection, compatible with contrast dye, and available in hypo‐ and hyperbaric liquid form, phenol might be preferable as a neurolytic agent for specific techniques [10]. When a hypobaric solution is needed—for instance, in intrathecal administration for patients unable to be positioned on the affected side—ethanol may be preferred, as its intrathecal use is associated with fewer side effects than phenol [45].

Given the risk of prolonged weakness, caution is advised when determining the location and dosage of phenol for nerves with both sensory and motor components [128]. Phenol not only induces motor weakness but also alters pain perception and causes numbness. This increases the risk of soft tissue injuries, including pressure or decubitus ulcers, necessitating careful monitoring and preventive measures [129].

For some adverse events, it is difficult to differentiate between the toxicity of phenol and its effects on the targeted nerves. For example, hypotension may indicate systemic toxicity or result from vasodilation during sympathetic nerve blocks. Potential risks, such as motor impairment from neurolysis, should be considered for the targeted nerves and adjacent structures due to potential phenol spread [130, 131]. When spread to spinal nerves is possible while targeting peripheral nerves, phenol in glycerol might be more suitable, and alternative techniques for neurolysis should be considered [132].

Elimination of phenol is theoretically prolonged in patients with end‐stage renal failure, as 60% is excreted via the kidneys. Due to the low systemic concentrations, a single administration is not expected to have clinical consequences. When systemic toxicity occurs, dialysis will not be effective due to the fast distribution of phenol.

## 7 Future Perspectives and Areas Requiring Further Research

Over the years, chemical neurolysis has expanded to chronic pain syndromes, and the range of therapeutic targets has broadened—including peripheral nerves, sympathetic nerves, and joints. Ultrasound has improved the safety of peripheral nerve blocks by enabling real‐time imaging and more precise nerve targeting, potentially supporting the safe use of phenol by experienced pain physicians.

Several other chronic pain syndromes may also warrant consideration for phenol neurolysis, as short‐term nerve blocks, often augmented with corticosteroids, can yield significant analgesic benefits in specific patient populations. For example, chronic pancreatitis is a complex problem with a high burden and substantial opioid use, which might justify more invasive interventions, such as celiac plexus neurolysis or splanchnic nerve neurolysis [100, 133]. Additionally, patients experiencing upper abdominal pain of visceral origin, such as pain from adhesions or inflammatory bowel disease, may benefit from neurolytic treatment [134]. Neurolytic block of the superior hypogastric plexus may help treat visceral pelvic pain arising from conditions such as endometriosis, persistent cystitis, pelvic inflammatory disease, or (postoperative) adhesions. Injection of bupivacaine in these pain syndromes showed effective but short pain relief [135]. A neurolytic block of the ganglion of Impar could be beneficial in patients with chronic pelvic pain mediated by sympathetic fibers of the perineum, rectum, and genitalia [136]. However, phenol neurolysis is not routinely recommended for chronic pain syndromes, and should be carefully weighed against the risk–benefit profile and the anticipated duration of the analgesic effect. Thermal ablation techniques might be an alternative when neurolysis is considered.

More recently, phenol neurolysis has been studied for various joint pathologies with promising results, including refractory knee pain, shoulder osteoarthritis, femoral fractures, and hip cancer [15, 110, 115, 137, 138]. Thermal ablation for hip capsule denervation is used in patients with coxarthrosis or persistent pain after total hip arthroplasty with variable results [139]. Several studies investigated the effect of chemical denervation of the hip capsule using ethanol in patients with hip pain due to fractures, osteoarthritis, or avascular necrosis. No neurological deficits or other adverse events were reported in these studies [93, 94, 140, 141].

Phenol may offer a reliable alternative to ethanol for this indication, as it can be administered without prior local anesthetics, avoiding dilution of its neurolytic properties. A retrospective study on chemical hip neurolysis with phenol did not report any complications in a cohort of 185 patients with a hip fracture, although the follow‐up period was limited [110]. For joint denervation, the volume of phenol should be kept low to minimize the risk of inadvertent spread to adjacent motor nerves [115, 142].

These potential targets and the increasing use of phenol highlight the need for additional well‐designed studies to understand the optimal target locations, dosage, risks, and patient selection criteria.

Although an established clinical utility, research on the use of phenol for specific interventions remains sparse, resulting in an absence of validated dosage guidelines. The long‐lasting nature of neurolytic procedures for a selected and often palliative patient population contributes to this sparsity. Nonetheless, phenol neurolysis holds considerable potential for innovation, particularly for peripheral nerves that are refractory to conventional thermal ablation methods. To advance current understanding, additional studies—including PK/PD studies, larger case series, or even randomized trials—are essential. In addition, implications of the solvent—water or glycerol—need to be elucidated to fine‐tune dosing regimens further. Furthermore, global practices in phenol administration vary widely, which can be attributed to limited access to phenol in certain institutions, a lack of clinical knowledge regarding its use, and concerns about potential adverse effects [6]. Further research and standardized guidelines may facilitate the development of optimized analgesic strategies for these patients.

## Author Contributions

J.C., R.J.H.S., R.D., and J.M.B.: conceptualized this study. J.H.A.C. and R.J.H.S.: wrote the manuscript. All authors revisited the manuscript critically for important intellectual content and approved the final version of this manuscript.

## Funding

The authors have nothing to report.

## Ethics Statement

The authors have nothing to report.

## Consent

The authors have nothing to report.

## Conflicts of Interest

The authors declare no conflicts of interest.

## Supporting information


**Box S1.** Overview of the implications of the use of phenol in clinical practice.

## Data Availability

The authors have nothing to report.
