# Supplementary material for: Phenol Neurolysis in Pain and Palliative Medicine
Source: Pain Pract. 2026 Apr 13;26(5):e70153. doi: 10.1111/papr.70153 (PMC13077028; doi:10.1111/papr.70153)
Supplement: Supplementary file 1 — Box S1. Overview of the implications of the use of phenol in clinical practice. [file PAPR-26-0-s001.pdf]

Supplemental box 1. Implications for clinical practice

Preparation

GMP-grade phenol solutions for clinical use do not require additional preparation. Phenol vapor does not have a substantial risk for bystanders.

Storage

Aqueous phenol solution (6%; 60mg/mL) and phenol in 85% glycerol (phenol 60mg/ml) should be stored at room temperature (15–25°C), protected from light, in an airtight glass container.

Precaution

Even phenol at low concentrations can cause burns after prolonged contact. Basic protective measures include the use of eyewear and gloves. Use dry bandages to protect the injection site.

Systemic reaction

Systemic reactions occur within minutes to hours. Cardiovascular, respiratory, and nerve system symptoms should be treated symptomatically according to local guidelines. No antidote is known and dialysis is not effective.

Corrosion

The risk of tissue damage is low if phenol is removed shortly after exposure. Polyethylene glycol should be used to remove phenol from the skin to minimize absorption. If polyethylene glycol is unavailable, rinse directly with large amounts of water when contacting the skin, eyes or mucous membranes for 15 minutes.
